# A Consolidated Review of Contemporary Targeted and Immunotherapeutic Options for Melanoma

**DOI:** 10.3390/biomedicines13061388

**Published:** 2025-06-05

**Authors:** Parker J. Champion, Jacob R. Bluestein, Anthony E. Quinn, Scott D. Bell, Josiah H. Kiley, Mark R. Wakefield, Yujiang Fang

**Affiliations:** 1 Department of Microbiology, Immunology & Pathology, Des Moines University, West Des Moines, IA 50266, USA; parker.champion@dmu.edu (P.J.C.); jacob.bluestein@dmu.edu (J.R.B.); anthony.e.quinn@dmu.edu (A.E.Q.); scott.d.bell@dmu.edu (S.D.B.); 2 Department of Surgery, University of Missouri School of Medicine, Columbia, MO 65212, USA; jhkwfn@missouri.edu (J.H.K.); wakefieldmr@health.missouri.edu (M.R.W.); 3 Ellis Fischel Cancer Center, University of Missouri School of Medicine, Columbia, MO 65212, USA

**Keywords:** melanoma, cancer, immunotherapy

## Abstract

The incidence of melanoma is increasing globally, even in the wake of increased risk factor awareness and a growing body of advanced therapeutic options. It is apparent that the treatment of melanoma will remain a topic of worry in areas of the world under high ultraviolet exposure and areas that harbor individuals with fair skin phenotypes. In the wake of such concern, the potential of immunotherapy and various targeted therapeutics to treat late-stage melanoma is increasing. In addition to the growing arsenal of PD-1 and PD-L1 immune checkpoint inhibitors, other targeted therapies are being developed and tested to treat melanoma. BRAF/MEK inhibitors target a key proliferative pathway in melanoma, offering clinical benefit but limited durability. Next-generation agents and triplet therapy with immunotherapy aim to improve outcomes. Androgen receptor signaling may also modulate responses to both targeted and immune-based treatments. Bispecific T cell engagers assist with guiding the body’s own T cells to tumors where they release toxins that kill the tumor cell. Personalized neoantigen vaccines target tumor-specific antigens by sequencing a patient’s cancerous cells to create tailored vaccines that elicit a strong and specific immune response. Tumor-infiltrating lymphocytes are autologous lymphocytes reinfused back into the host that are showing efficacy in the treatment of advanced melanoma. Together, these therapies are advancing the arsenal of chemotherapeutic options that can be used to inhibit the progression of melanoma.

## 1. Introduction

Melanoma is a malignancy developing from mutated melanocytes responsible for producing pigment. While the cutaneous form is the most common, malignancies can arise in other areas [1,2]. Melanoma is considered the deadliest skin cancer; this is attributable to its high propensity to metastasize [3]. The 5-year relative survival rate for localized melanoma is nearly 100% but decreases to around 34.6% for cases of melanoma with distant metastases [4]. In earlier stages, surgical excision is effective, but the prognosis of patients with later-stage disease is poor with conventional methods [5]. Globally, the rate of melanoma is rising; between 1990 and 2019, the incidence of melanoma is estimated to have increased by 170% [6]. In 2020, an estimated 325,000 new melanoma cases were diagnosed worldwide, and 57,000 deaths were attributed to melanoma. If these rates continue, new melanoma cases and deaths are projected to reach 510,000 and 96,000, respectively, by the year 2040 [7]. Traditional chemotherapy, such as dacarbazine, has a response rate of only around 15%. Targeted therapies have drastically improved survival for patients with advanced, BRAF-mutated melanoma, especially when compared to traditional chemotherapy. Moreover, immunotherapies have demonstrated outstanding advancement in progression-free survival (PFS) and overall survival (OS), regardless of their mutation characteristics [8,9,10].

Immune checkpoint inhibitors (ICIs) are a standard of therapeutic intervention for metastatic melanoma [11]. Anti-CTLA-4 agents were the first ICIs that were considered effective for cancer treatment, and ipilimumab was the first approved anti-CTLA-4 monoclonal antibody (mAb) for metastatic melanoma [11]. PD-1 is an inhibitor of immune activity that is largely expressed on tumor-specific T cells. PD-L1, a ligand of PD-1, also inhibits the function of anti-tumor immune cells, and is present in high numbers in most cancer cells [12]. Therapies with the mAbs nivolumab and pembrolizumab are effective PD-1 inhibitors that promote the destruction of cancer by increasing the immune response [13]. A phase III clinical trial (CheckMate 066) demonstrated that drugs such as nivolumab demonstrate a superior increase in five-year overall survival, PFS, and an overall response rate when compared to the standard chemotherapeutic drug dacarbazine [14]. Another notable phase 3 clinical trial (KEYNOTE-006) showed that the PD-1 inhibitor pembrolizumab improved survival in comparison to the anti CTLA-4 immune checkpoint inhibitor, ipilimumab [15]. These foundational studies cement PD-1/PD-L1 inhibitors as a first-line treatment for melanoma, but there are remaining challenges such as primary and acquired resistance [16], underscoring the need for continued research.

## 2. BRAF-MEK Inhibitor Combination Therapy

**Figure 1:**
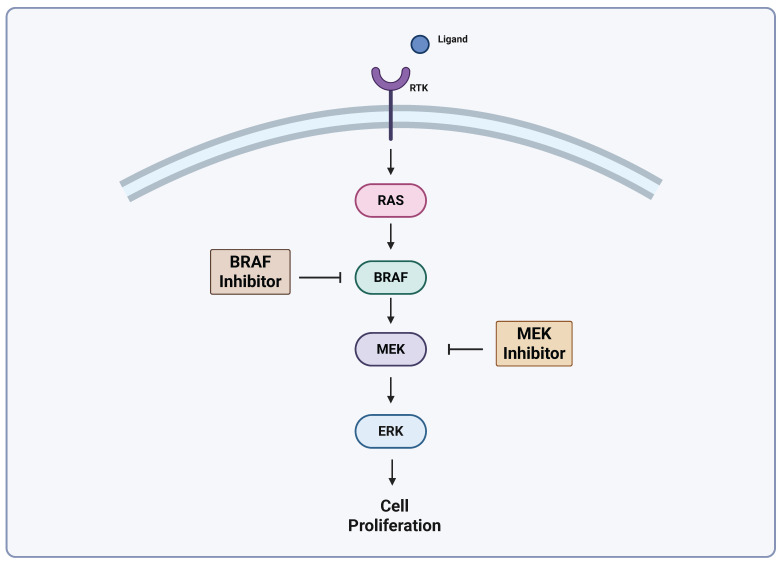
Simplified MAPK pathway. Adapted from Castellani et al. [25], Lelliot et al. [26]. Created with biorender.com (https://app.biorender.com/illustrations/680aa2b3ad24390f362a14b2?slideId=c338376b-1ff2-473a-a8cf-cf87ba6d7336, accessed on 24 April 2025).

The Mitogen-Activated Protein Kinase (MAPK) pathway is central to the pathogenesis of melanoma and activating mutations in this signaling cascade lead to proliferation signals in cancer cells [17]. Ligand engagement with a receptor tyrosine kinase (RTK) activates RAS, a GTPase that functions upstream of the well-characterized MAPK pathway kinases: RAF, MEK, and ERK [18,19]. A simplified MAPK pathway is shown in Figure 1. Mutations of this cascade are highly prevalent in human cancer, and the isoform of RAF known as BRAF is implicated in as many as 60% of melanomas [19,20]. The advent of drugs targeting this pathway has led to significant improvements in cancer treatment. BRAF inhibitors (BRAFi) are effective, but resistance is a problem, and results with BRAFi monotherapy alone do not produce durable responses [21]. It has been shown that combined BRAF/MEK inhibition significantly improve OS and PFS versus BRAF inhibition alone. There are currently three FDA-approved BRAF/MEK inhibitor combinations approved for advanced melanomas [22,23]. Combined BRAF/MEK inhibition has become the standard of care for melanomas with BRAF mutations, but improvements are necessary to combat acquired resistance. Even with the success of these drugs, responses are temporary, and relapse occurs in many patients [21]. Targeted therapy such as BRAF/MEK inhibition is showing promise when combined with ICIs [24].

Multiple mechanisms of resistance have been studied, but most involve the paradoxical reactivation of the MAPK pathway. Resistance to first-generation BRAFis vemurafenib and dabrafenib can be explained by aberrations causing the dimerization of BRAF, leading to downstream ERK activation [27]. So-called “paradox breakers” such as PLX7904 and its more optimized analogue PLX8394 were developed as structural variants of vemurafenib designed to avoid MAPK pathway hyperactivation in wild-type cells. PLX8394 is a novel BRAFi that does not induce the paradoxical activation of MAPK. It inhibits ERK via selective RAF inhibition by targeting oncogenic BRAF dimers while preserving RAF activity in normal cells. These characteristics support its potential safety and effectiveness in treating tumors harboring BRAF mutations [28]. A phase 1/2 trial (NCT02428712) evaluating PLX8934(FORE8394) demonstrated antitumor effects without paradoxical reactivation in varied tumor types, including melanoma. Included in this study were patients previously exposed to MAPK inhibitors [29]. These findings present a strong rationale for the continued investigation of PLX8934.

PF07799933, a selective, next-generation inhibitor of BRAF, has been designed to inhibit a larger subset of BRAF mutations, including dimer and non-dimer variants, a property that is unique when compared to first-generation BRAF inhibitors. Brain-penetrant properties have also been identified, making it an ideal candidate for the treatment of intracranial metastases [30,31]. A first-in-human phase 1 trial (NCT05538130) is currently underway. It uses a novel dose escalation design to investigate the safety, pharmacokinetics, pharmacodynamics, and anti-tumor activity in patients with advanced disease who have progressed on first-line BRAF/MEK inhibitors [32]. Thus far, PF-07799933 has been well tolerated as both monotherapy and combination therapy for melanoma and elicited tumor reduction in patients who were treated with BRAF inhibitors in the past. Future direction with this drug will likely include a MEK inhibitor as an adjuvant [30].

In addition to the development of next-generation BRAF and MEK inhibitors (MEKi), efforts are underway to combine these agents with immunotherapy. This is based in part on preclinical evidence suggesting that BRAF and MEK inhibition can modulate the tumor immune environment. Sumimoto et al. observed a decrease in immunosuppressive factors after a BRAF-mutated cell line was treated with a MEKi. It is postulated that phosphorylated ERK may induce a reduction in immune factors [33]. In a trial involving patients with metastatic melanoma, treatment with either a BRAF inhibitor (vemurafenib) or a BRAF/MEK inhibitor combination (dabrafenib and trametinib) experienced a significant increase in CD8^+^ T lymphocyte infiltration, as confirmed by tumor biopsy [34].

Selective BRAFi, PLX4720, elicited increased antigen expression, specifically in BRAF-mutated cell lines. MAPK pathway inhibition was found to upregulate melanocyte differentiation antigens (MDAs) expression and improve recognition by antigen-specific T cells. These antigens represent key targets for cytotoxic T cell-mediated tumor recognition. This study also evaluated two selective MEKis, U0126 and PD0325901, and MDA expression was noted to be increased regardless of BRAF mutation status. Notably, however, these data also demonstrated that the inhibition of MEK impairs T lymphocyte action, whereas BRAF inhibition does not [35]. Furthermore, BRAF inhibition with dabrafenib and MEK inhibition with trametinib has been identified as increasing PD-1 expression in vitro. The BRAF inhibitor vemurafenib increases MHC class I and II expression. These effects support immune checkpoint inhibition as a useful addition to metastatic melanoma therapy [2,34,36].

Building on preclinical and early clinical findings, several trials have attempted to assess the combination of BRAF and MEK inhibition with immune checkpoint blockade, referred to as triplet therapy. Most notably, one such study led to FDA approval. IMspire150 (NCT02908672) was a randomized, double-blind, placebo-controlled phase 3 clinical trial that assessed vemurafenib and cobimetinib with or without atezolizumab in patients with metastatic BRAF-mutated melanoma. As assessed by the investigator, PFS was determined to be significantly longer in the atezolizumab group (15.1 months vs. 10.6 months in the control group). However, an independent review committee found an increase in PFS that did not meet the threshold for statistical significance (16.1 months vs. 12.3 months). Adverse effects were seen more commonly in the treatment group, but 3% more patients dropped out in the control group due to adverse effects. This study not only demonstrated an increase in PFS but also acceptable tolerability [24]. Based on these findings, the triplet combination received FDA approval [24,37]. Long-term follow-up with IMspire150 showed that, despite maintaining the PFS benefit, there was no statistically significant OS benefit [38]. The positive results and subsequent FDA approval demonstrate that triplet therapy is clinically feasible. Although long-term follow-up diminished the perceived impact of the study, the further investigation of similar combinations remains warranted.

Another recent phase 3 study, COMBI-i (NCT02967692), investigated a similar but distinct triplet regimen combining spartalizumab (PD-1 blockade) with dabrafenib and trametinib. The combination did show improvement in the median progression-free survival (16.2 vs. 12.0 months), but did not meet its primary endpoint. These findings reflect the difficulty in achieving consistent benefit from triplet therapy, despite encouraging preclinical data [39,40]. Even without meeting a primary endpoint, significant data suggest a synergistic effect of MAPK inhibition and immunotherapy. Based on these two notable clinical trials, triplet therapy shows great promise for the treatment of advanced melanoma. However, based on the inconsistency of results, it is clear that work needs to be undertaken to optimize future treatment regimens, and patient populations.

A consistent female survival advantage has been observed in melanoma across multiple studies, with women demonstrating better outcomes than men, even after adjusting for tumor and patient characteristics [41]. Recent studies demonstrate that androgen receptor (AR) activity contributes to resistance against immunotherapy in melanoma. The inhibition of AR signaling has been shown to enhance the response to immune checkpoint inhibitors [42]. AR-positive melanoma is linked to worse clinical outcomes, with androgen receptor signaling promoting tumor progression through the regulation of invasion and metastasis related pathways [43]. While the mechanism of AR’s observed effects is not entirely understood, recent data show that AR signaling enhances melanoma invasiveness by upregulating fucosyltransferase 4 (FUT4), leading to the dysregulation of cellular junctions. The fucosylation of the L1 cell adhesion molecule (L1CAM) by AR-activated FUT4 further promotes metastatic progression in melanoma [44]. Evidence of immune modulation by AR activation supports a rationale for combination therapy with immunotherapy. No clinical data have been produced on the topic but their existence is plausible based on pre-clinical information.

An analysis of patients enrolled in a clinical trial (NCT02231775) investigating BRAF/MEK therapy demonstrated a significantly improved 2-year recurrence-free survival rate in female patients compared to males (64% vs. 32%). The authors conducted translational studies using preclinical murine models to explore potential mechanisms underlying this sex-based disparity. Androgen receptor (AR) expression was elevated in both male and female mice following BRAF/MEK-targeted therapy. The pharmacologic inhibition of AR signaling improved therapeutic responses in both sexes, whereas testosterone administration impaired the response to BRAF/MEK inhibition in male and female mice [45,46]. While sex-based differences in immune responses have been clinically observed, recent evidence suggests that sexual dimorphism alone does not account for disparities in melanoma outcomes. AR signaling promotes tumor progression and impairs antitumor immune function in both male and female melanoma cells. This evidence supports the role of AR as a broadly relevant therapeutic target in melanoma, with potential utility beyond patient sex-based differences [47]. This recent body of work further supports evidence that biological sex-based differences play a role not only in cancer prognosis but also in the efficacy of BRAF/MEK-targeted therapies. Patient sex should be considered when designing treatment regimens for melanoma. Despite preclinical support, there are currently no clinical trials investigating BRAF/MEK inhibition in combination with the AR blockade.

## 3. Bispecific T Cell Engagers

Bispecific T cell engagers (TCEs) are a recent and novel approach to fighting tumors using local T cell recruitment. The bispecific antibody binds to a tumor cell with its Tumor-Associated Antigen (TAA) domain, while its other domain traditionally binds to a CD3 T cell receptor. The bispecific nature allows T cells to be led close to tumor cells, where the T cell can be activated with the CD3 domain to release perforin and granzyme, killing the tumor cell. Current TCE development is focused on the specific TAA targeted, which allows for different tumor cells to be targeted based on the TAAs they display. In addition, some newer developments explore the possibility of using a different T cell receptor or injecting specific cytokines to improve the TCE immunological outcome.

**Figure 2:**
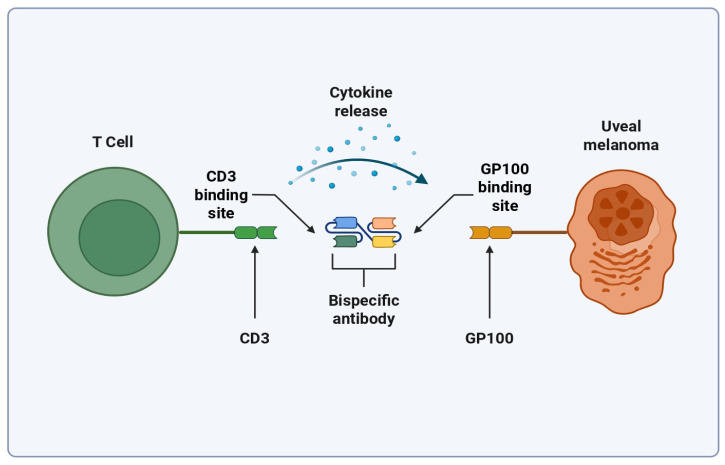
Simplified mechanism of Tebentafusp binding. Created with biorender.com (https://app.biorender.com/illustrations/6807a7148f6f387ae6e098af?slideId=00d55f78-9ce9-49fe-8955-ebcaa3537b15, accessed on 25 April 2025).

Tebentafusp is a bispecific T cell-engaging antibody designed to attach to Glycoprotein-100 (GP100) molecules displayed on Human Leukocyte Antigen-A0201 (HLA-A0201) on the surface of uveal melanoma cells, where this molecule is commonly found [48]. Figure 2 shows an illustration of this binding. Variability in the expression of GP100 in primary and metastatic melanomas exists, although it was found that it does not change the outcome of Tebentafusp [49,50]. A phase three trial was completed in 2021 which showed that Tebentafusp markedly improved outcomes in those with the disease (NCT03070392). It was found that the one-year survival rate for patients taking Tebentafusp to be 73%, up from 59% in the control group (*p* < 0.001) [48]. This led to the FDA approval of the drug in January of 2022, making it the first and only TCE to be approved for use with melanoma [51]. A study completed in 2022 further showed that Tebentafusp improves patient outcomes in patients with previously treated metastatic uveal melanoma, widening the scope of the treatment’s usage (NCT02570308) [52]. A common complication of Tebentafusp and other TCEs is the development of Cytokine Release Syndrome (CRS), a currently poorly understood complication that results in a large increase in a wide variety of cytokines [53]. In addition, 83% percent of patients who receive Tebentafusp have adverse cutaneous reactions, likely because of the gp100 expression in melanocytes [54,55]. A study suggests that Tebentafusp may be modifying the tumor microenvironment (TME) to favor metastatic melanoma outcomes by increasing IL-7R gene expression in memory and naive T cells, and by increasing local Interferon (IFN) signaling [50,56,57]. Cells exposed to IFN-γ have been found to be more sensitive to cytotoxic T cell attacks through the upregulation of molecules associated with major histocompatibility complex (MHC) class 1. Also of note is that CD4^+^ and CD8^+^ T cells make a positive feedback loop with the IFN-γ they release, and that natural killer (NK) cells provide the main innate immunity production of IFN-γ [58].

With Tebentafusp being the main TCE treatment option for patients with melanoma, factors surrounding its use are being analyzed to further our understanding of its mechanism and effectiveness. A quantitative systems pharmacology model was used to identify predictive biomarkers to distinguish responders and non-responders before treatment is given. It was found that CD4^+^ and CD8^+^ density and the pre-treatment CD8^+^/Treg cell ratio can be predictive of whether a patient will respond to treatment [59]. Tumor-associated macrophages (TAMs) have been shown to be predictive of Tebentafusp outcomes as well. A study suggests that combining IL-2 with Tebentafusp might reduce tumor macrophage-caused suppression and assist in treatment outcomes in cases with high TAM-to-T cell ratios [60,61]. A separate study also found that increased IFN signaling in the tumor at baseline is associated with a better result from Tebentafusp (NCT02570308) [52]. Another important factor to consider is that natural killer cells play a significant role in the initial immune response to melanoma, and a varied role later in the disease progression. Certain TAMs can lead to NK cells being suppressed or filling an immune-suppressive role depending on the cytokines produced [62,63]. NK-focused solutions alongside Tebentafusp could represent a promising field of study for melanoma treatment. PD-1/PD-L1 checkpoint inhibitors have shown promising results for melanoma therapy by binding and inhibiting PD-1 receptors expressed on cancer cells, reducing tumor evasion and immune downregulation. In addition, PD-1 checkpoint inhibitors have been found to increase IFN-γ levels and increase cytotoxic response against the tumor [64,65]. A phase one trial found that Tebentafusp and durvalumab, an anti-PDL1 treatment, was found to be safe and show efficacy, signaling promise for a combination treatment for patients with uveal melanoma (NCT02535078) [66]. A separate study found that PD-1/PD-L1 checkpoint inhibitors are not very effective in uveal melanoma by themselves (NCT01355120) [67]. A phase three trial comparing Tebentafusp treatment with and without a PD-1 inhibitor is currently recruiting and is set to finish in 2028, which will elaborate on this aspect of potential treatment (NCT05549297). Studies have not yet elaborated on any downregulation of GP100 or PD-L1 in response to TCEs, which remains an unknown.

Developments in the Melanoma TCE field separate from Tebentafusp also exist. A CD3 x PD-L1 nanobody was designed as a potential novel TCE, intended for targeting melanoma with increased PD-L1 expression [68]. More recently a study verified the efficacy for CD3 x PD-L1 bispecific T cell engagers that are pre-bound to T cells. Their results showed that the migration of T cells to the tumor site was closely related to the expression of PD-L1 protein, the cancer cell surface target of BsTE molecules, and PD-L1-containing exosomes secreted by cancer cells. Additionally, it was found that, in murine B16 melanoma, there was superior tumor elimination relative to T cells or the TCE molecule alone [69]. A patent for TCEs targeting human MET gene expression was also submitted, although it was rejected [70].

## 4. Personalized Neoantigen Vaccines

TAAs have been an important component of cancer therapy for decades and continue to yield strong therapeutic outcomes, particularly with TCEs. However, recent research has started to utilize tumor-specific antigens (TSAs) or neoantigens [71]. TSAs are unique because they target antigens found exclusively on cancer cells, rather than broadly overexpressed tumor markers (TAAs), which are less likely to provoke a distinct immune response [72]. TSAs are the result of continuous divisions in cancer cells leading to specific mutated antigens through genomic mutations such as dysregulated RNA splicing, post-translational modifications, or integrated viral sequences [73].

Personalized neoantigen vaccines (PNVs) represent a novel form of immunotherapy. PNVs utilize patient-specific TSAs, identified through next-generation sequencing and computational modeling, like artificial intelligence (AI), to generate vaccines aimed at priming T cells against tumor-specific epitopes. The process begins with DNA extraction from tumor and normal cells, followed by the identification and validation of immunogenic neoantigens, and ends in vaccine formulation with adjuvants such as CAF^®^09b or viral vectors [74]. These vaccines are particularly well suited for melanoma due to the high mutational burden of the disease, which provides a large source of immunogenic neoantigens [75].

**Figure 3:**
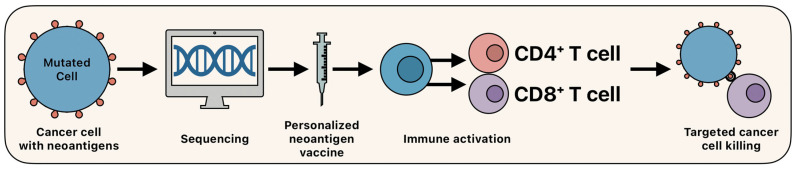
Overview of personalized neoantigen vaccines.

The development of PNVs involves several stages shown in Figure 3. Tumor-specific mutations are identified via whole-exome or RNA sequencing, followed by epitope prediction using MHC-binding algorithms. Candidate neoantigens are then synthesized and formulated into a vaccine and delivered either intradermally, via intranodal injection, or orally [74,76]. These vaccines function by activating dendritic cells and priming cytotoxic and helper T lymphocytes. Unlike TAAs, neoantigen vaccines elicit highly specific responses, minimizing off-target effects and reducing immune tolerance [77].

As the clinical interest and feasibility of PNVs expands, a growing number of early-phase trials are demonstrating their safety, feasibility, and potential in the treatment of melanoma.

In a phase I study by Mork et al., five patients with metastatic or unrespectable melanoma were started on ICIs at the time of biopsy [78]. After 6–8 weeks, five patients were vaccinated with an EVX-01 personalized neopeptide along with the CAF^®^09b adjuvant, a liposomal adjuvant composed of dimethyldioctadecylammonium bromide and C-type lectin receptor agonist monomycoloyl glycerol (MMG), and TLR3 agonist Poly I:C. CAF^®^09b enhances the uptake by dendritic cells and activates innate immunity through TLR2 and TLR3 pathways, promoting strong CD4^+^ and CD8^+^ T cell priming [79]. All patients from the study were able to elicit an immune response with only non-severe side effects including fatigue and pain at injection site [78]. Of the five patients, one patient had a complete response, two patients had a partial response, and two patients progressed. The vaccine was well tolerated, with only mild adverse events reported. This study demonstrates the feasibility of creating safe and effective neoantigen vaccines.

Another trial conducted by D’Alise, et al. tested a viral vector–based neoantigen vaccine named NOUS-PEV. This phase I study enrolled six patients with unresectable or metastatic melanoma. The vaccine was engineered to encode 60 tumor-specific neoantigens using a heterologous viral prime-boost system, consisting of a GAd20 adenoviral vector primer and a Modified Vaccinia Ankara booster, combined with pembrolizumab. Within 8 weeks of biopsy collection, vaccinations were administered with four out of six patients eligible to receive the full combination. All vaccinated patients were able to elicit a long-lasting immune response with cellular immunity still detected 7 months after vaccination with no serious adverse events. Of the 6 patients vaccinated there was one complete response, three partial responses, one stable disease and one disease progression [80]. Overall, this study showed that viral vector based neoantigens can create strong immunity in some patients with melanoma.

The study by Vaitiekus et al. focused on NECVAX-NEO1, a personalized DNA-based neoantigen vaccine. The trial enrolled five patients with melanoma, renal cell carcinoma, or head and neck cancer. Neoantigens were encoded into a DNA construct and delivered orally. Of the neoantigens encoded, 68% induced a significant immune system response measured by ELIspot. All patients experienced an immune response to the target antigens after vaccination, with two out of five patients experiencing a significant rise in neoantigen signaling. Four patients achieved stable disease at 24 weeks. No vaccine-related toxicities were observed [81]. This study demonstrated both the efficacy and safety of oral DNA vaccines in solid tumors.

KEYNOTE-942 is a phase II personalized neoantigen mRNA vaccine and anti-PD-1 combination therapy study in patients with resected stage III melanoma. In this study, 50 patients received monotherapy with pembrolizumab, while 37 received combination therapy with personalized mRNA vaccines containing 9–34 unique epitopes. Combination therapy was initially planned to have 107 participants but was condensed due to manufacturing limitations during the COVID-19 pandemic. After a median 23-month follow-up, relapse-free survival was longer for patients treated with combination therapy, with a 44% reduced risk of relapse or death compared to monotherapy. However, these results were not statistically significant (*p* = 0.561), presumably due to the small sample size. The distant metastasis-free survival rate also favored the combination therapy with few grade 1–2 side effects (fatigue, chills, injection site pain). Additionally, researchers found that circulating tumor DNA (ctDNA) negative patients had a lower recurrence rate of 17% compared to 94% in ctDNA-positive patients [82]. This study suggests that mRNA vaccines can enhance current immunotherapies with a few mild side effects.

These studies highlight the various ways to produce personalized cancer vaccines, including the use of peptides, DNA, mRNA, or viral vectors. Even though the methods and delivery systems differ, they all have the same central purpose: to induce specific T cell responses that target the specific mutations of each patient’s tumor.

## 5. Tumor-Infiltrating Lymphocytes

Melanoma traditionally has a high tumor mutational burden corresponding to an increased number of tumor neoantigens [83]. These enhanced neoantigen levels make melanoma a prime target for immune therapies that utilize the host’s own immune response due to its ability to recognize and destroy neoantigen-presenting cells [84]. Tumor-infiltrating lymphocyte (TIL) therapy involves the excision of TILs from the tumor microenvironment followed by in vitro expansion and infusion back to the patient [85]. The effectiveness of TILs directly correlates to the extent of tumor infiltration and involvement, thus ensuring that TILs can infiltrate the tumor microenvironment effectively remains central to their effectiveness [86].

Classically, TILs have been used as an indicator of immune system response to melanoma and a positive predictor of response to treatment [87]. TILs typically consist of CD4 T cells that can recognize antigens presented on MHC class II molecules that present exogenous antigens, and CD8 T cells (CTLs) that recognize MHC class I molecules that present endogenous antigens. Both subtypes can elicit an immune response to the tumor and have been correlated with positive responses to melanoma tumor therapy [88]. TILs have shown efficacy in eradicating metastatic melanoma, even more than some previous therapeutic regimens such as various ipilimumab therapy plans or systemic IL-2 treatment in some cases [89]. Besides their use as prognostic indicators of immunotherapy progression, TILs have recently been receiving increased attention regarding their use as a stand-alone therapy for advanced melanoma [90].

**Figure 4:**
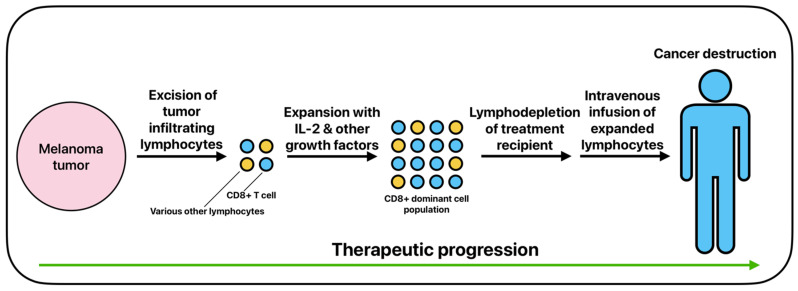
The basic process of adoptive cell therapy using tumor-infiltrating lymphocytes.

TIL therapy for melanoma generally involves excising a portion of the melanoma tumor, expanding TILs that show the tumor killing response in vitro with IL-2 and other growth factors, and intravenously infusing the TILs back into the patient (Figure 4) [91,92]. TILs will generally be selected for by CD8 positivity after expansion due to CD8 positivity indicating proven response rates in cancer destruction [93,94]. Prior to post-expansional TIL infusion, patients will typically be administered a non-myeloablative lymphodepletion regimen that will allow the infused TILs to take full precedence in immune response to the targeted tumor [85]. Lymphodepletion is usually achieved via chemotherapy regimens or total body irradiation, thus allowing the downregulation of regulatory T cells and an upregulation of cytokines that promote positive treatment response [85,95,96].

In 2024, the FDA approved the use of TILs for use in patients with treatment-resistant, refractory metastatic melanoma. This FDA approval has opened the door for TIL therapy as a feasible treatment method in certain advanced cases of melanoma. TIL therapy has been in refinement for over three decades, with Rosenberg et al. first describing the use of TILs being expanded ex vivo and then reinfused with systemic IL-2 for the treatment of metastatic melanoma [97]. Recently, various studies have shown efficacy in treating melanoma with TILs. Rohaan et al. investigated the infusion of at least 5 × 10^9^ TILs into 84 patients against 84 other patients treated with ipilimumab mAb, all with unresectable stage IIIC or IV melanoma [90]. In the intention-to-treat population, median PFS was 7.2 months in the TIL group and 3.1 months in the ipilimumab group [90]. Furthermore, median overall survival was found to be 25.8 months in the TIL group and 18.9 months in the ipilimumab group [90]. This study was important in highlighting the use of TIL therapy in advanced melanoma as it directly compared the use of TILs to traditional mAb therapy, with TILs showing superior efficacy in the study.

Pilon-Thomas et al. administered a complete treatment consisting of TILs with IL-2 post-non-myeloablative chemotherapy to 13 patients with metastatic melanoma. Of the patients, two showed complete response to treatment, three showed partial response to treatment, and four showed a stable disease ranging from 2 to 24 months. The group noted that the treatment was laborious to instill but showed a promising response rate [98]. In a phase II clinical trial, Nguyen et al. enrolled 12 patients with metastatic melanoma and treated them with autologous TILs and low-dose subcutaneous IL-2 following non-myeloablative chemotherapy. Of the patient cohort, two patients showed a partial response, one patient showed an unconfirmed partial response, and six patients showed stable disease [99]. Nguyen et al. concluded that their TIL treatment regimen proved clinical feasibility and efficacy in the treatment of metastatic melanoma [99]. Furthermore, Larkin et al. administered TIL therapy to patients with advanced unresectable and/or metastatic melanoma previously treated with anti-LAG3 antibody and ICIs. TIL therapy in this group gave an objective response rate of 38.5% and 60% of therapeutic responses were still found durable beyond 12 months [100]. Seitter et al. performed a retrospective analysis of patients with metastatic melanoma who were administered TILs with IL-2 following lymphodepletion. The treatment regimen gave an objective response rate of 56% and median melanoma-specific survival of 28.5 months in patients that had not been previously treated with anti-PD-L1 therapy. In comparison, the treatment regimen gave an objective response rate of 24% and median melanoma-specific survival of 11.6 months in patients that were refractory to anti-PD-L1 [101].

Moreover, many ongoing clinical trials are investigating ways to optimize TIL usage. The phase I clinical trial NCT05470283 is assessing the usage of TILs engineered with membrane bound IL-15 is being investigated with the aim to eliminate the use of IL-2. Thus far, nine patients with ICI-resistant metastatic melanoma have been treated with the IL-15 engineered TILs have shown no dose-limiting toxicities [102]. Furthermore, the ongoing phase 3 TILVANCE-301 study is comparing the usage of TILs with pembrolizumab versus pembrolizumab alone in treatment-naïve unresectable or metastatic melanoma [103]. The TILVANCE-301 study will help guide further direction in combination therapy for advanced melanoma with previous standards utilizing pembrolizumab. Another study investigating the use of TILs versus pembrolizumab is the phase 2 IOV-COM-202 study (NCT03645928). An early cohort of 22 patients from the IOV-COM-202 study with ICI-naive unresectable or metastatic melanoma who were treated with TILs and pembrolizumab showed positive efficacy in treatment response [104].

It is apparent that that TIL therapy will eventually play a major role in the treatment of advanced melanoma. With TIL therapy’s recent FDA approval, efficacy in the treatment of refractory melanoma, and increased interest in clinical trials, there is a high possibility that it could replace traditional treatment regimens. Specifically, the TILVANCE-301 study will aid in guiding where TIL therapy fits regarding standard of care therapies using ICIs such as pembrolizumab.

## 6. Conclusions

The emergence of targeted therapies and immunotherapies has led to significant advances in the treatment of metastatic melanoma. BRAF/MEK inhibition remains a cornerstone of targeted therapy for BRAF-mutated melanoma and continues to play a key role in frontline treatment. Next-generation inhibitors are being developed to overcome resistance and improve tolerability. Triplet therapy combining BRAF/MEK inhibition with immunotherapy has shown promise, though clinical outcomes remain variable and further optimization is needed. As these strategies evolve, the contribution of BRAF/MEK inhibitors to durable disease control will be better defined. AR signaling is also gaining attention in melanoma, with growing evidence linking AR activity to metastasis and resistance to both immunotherapy and targeted therapy. AR may represent a critical future target in the evolving landscape of melanoma treatment.

TCEs are a relatively new therapy focusing on localizing T cells to tumors by using an antibody that binds to both the T cell and a TAA on the tumor. The increased specificity of TAAs for treatment and ability to guide T cells where needed makes it a promising field to explore, but care needs to be taken to ensure that the treatment does not also over-target other cells that express the TAAs. As with Tebentafusp, slight differences in antigen presentation between cells can still mean that T cells will be led to the wrong cell for lysing, so care must be taken to ensure that potentially dangerous conditions, such as CRS, do not cause the treatment to inflict harm upon the patient. In addition, factors such as the changing tumor microenvironment and permeability to T cells remain important for treatment design and outcome.

PNVs are a step towards truly personalized healthcare for melanoma. DNA is taken from healthy and tumor cells, and valid immunogenic neoantigens are identified with computational modeling. These neoantigens are then used to design vaccines that prime the immune system to target the antigens on tumor cells. These vaccines can use a variety of immunogenic vectors, such as DNA, RNA, peptides, or viral vectors. In addition, the specificity of this method of melanoma targeting means that unintended immune responses to other tissues is minimized.

TIL therapy is currently leading the field of autologous cell therapies for melanoma. With treatment optimization occurring for over three decades now and recent FDA approval, TILs have the potential to play a major role in the treatment of advanced melanoma. At the conclusion of more trials examining TILs in comparison to previous standard of care mAb regimens, a more definitive answer can be given on their exact place in melanoma immunotherapy. However, results from recent completed and ongoing clinical trials are indicating greater efficacy than current standard-of-care mAb treatment regimens in various advanced melanoma settings.

## Data Availability

No new data were created or analyzed in this study. Data sharing is not applicable to this article.

## Abbreviations

The following abbreviations are used in this manuscript:

AR	Androgen receptor
BRAFi	BRAF inhibitor
CRS	Cytokine release syndrome
ctDNA	Circulating tumor DNA
CTLs	Cytotoxic T lymphocytes
FUT4	Fucosyltransferase 4
GP100	Glycoprotein-100
HLA	Human leukocyte antigen
ICI	Immune checkpoint inhibitor
IFN	Interferon
L1CAM	L1 cell adhesion molecule
mAb	Monoclonal antibody
MAPK	Mitogen-activated protein kinase
MDA	Melanocyte differentiation antigens
MEKi	MEK inhibitor
MHC	Major histocompatibility complex
OS	Overall survival
PFS	Progression free survival
PNV	Personalized neoantigen vaccine
RTK	Receptor tyrosine kinase
TAA	Tumor-associated antigen
TAM	Tumor-associated macrophages
TCE	Bispecific T cell engager
TIL	Tumor-infiltrating lymphocyte
TME	Tumor microenvironment
TSA	Tumor-specific antigen
